# Expansible thermal gelling foam aerosol for vaginal drug delivery

**DOI:** 10.1080/10717544.2017.1375575

**Published:** 2017-09-18

**Authors:** Liling Mei, Jintian Chen, Siqin Yu, Ying Huang, Yecheng Xie, Hui Wang, Xin Pan, Chuanbin Wu

**Affiliations:** School of Pharmaceutical Sciences, Sun Yat-sen University, Guangzhou, China

**Keywords:** Temperature dependent, vaginal drug delivery, vaginal infection, phase transition, poloxamer

## Abstract

Vaginal delivery of antimicrobial drugs is the most effective method for the local treatment of the vaginal infections. However, current vaginal drug delivery systems (VDDS), including gel, lotion, aerosol and cream, are suffering from low penetration in the deep vaginal rugae and easy elimination by self-cleaning of vaginal canal. To address these issues, a foam aerosol based on the thermal transformation was designed to improve penetration efficiency and achieve the extended retention. The expansible thermal gelling foam aerosol (ETGFA) consisting of thermal sensitive matrix, silver nanoparticle, adhesive agent and propellant, was optimized by evaluations of precursor viscosity, foam expansion, thermal gelation, gel adhesiveness, antimicrobial effects and tissue irritation. The ETGFA would penetrate to the deep vaginal rugae to cover the infectious sites by foam expansion. Drug leakage was intended to be avoided by the thermal gelation at physiological temperature before foam collapse. The gel could be retained in the vaginal canal for extended time due to its superior adhesiveness when compared to the commercial gel Asimi^®^. The ETGFA provided extended drug release for over 4 h and maintained effective drug concentrations at the infectious sites. The ETGFA containing silver nanoparticles showed dose-dependent antimicrobial effects on the vaginal floras and irritation reduction to the vaginal tissues. The results demonstrated that the ETGFA could overcome the limitations of conventional dosage forms, including poor drug penetration, carrier retention and patient compliance and satisfied the requirements for vaginal drug delivery.

## 1. Introduction

Vaginal infection is an increasingly prevalent gynecological disease, whose treatment requires effective concentrations of antimicrobial drugs at the infectious sites. The antimicrobial drugs are often administrated by oral, injection or vaginal drug delivery system (VDDS) (Li et al., 2012). The VDDS is regarded as the most effective method for the local treatment of vaginal infection, which could circumvent the first-pass effect metabolism and unnecessary drug distribution in the blood circulation after oral administration (Johal et al., 2016). Also, it could minimize administration inconvenience and painful feeling during injection. The VDDS could effectively offer stable drug concentrations for local treatment of vaginal infection and minimize the risk of irritation to the vaginal tissues.

Recently, a wide variety of dosage forms including lotion, aerosol, gel, suppository, cream and tablet, have been introduced for vaginal drug delivery. However, the efficacy of these dosage forms is dramatically limited by low penetration efficiency in the deep vaginal rugae and easy elimination by self-cleaning of vaginal canal. Therefore, penetration to the deep vaginal rugae and adhesion to the vaginal mucosa are the desirable properties for a favorable VDDS dosage form.

Foam aerosol has distinct superiorities for vaginal administration which include direct penetration to deep vagina rugae and convenience for self-administration (Junyaprasert et al., 2008). Also, the potential contamination of the remaining proportion of drug could be avoided as the drug is stored within and spurted out from a sealed container. However, there are some drawbacks such as short foam duration, rapid foam collapse and subsequent liquid leakage, resulting in poor therapeutic efficacy.

For effective therapy, multiple and frequent administrations are required to make compensation for the removal of drug carrier. It is reported that prolonged carrier retention and extended drug release could be achieved by gel because of its better mucosal adhesion and superior gel strength (Mei et al., 2017; Timur et al., 2017; Tugcu-Demiroz, 2017). However, the application of gel in vaginal drug delivery is limited by its low penetration efficiency to the deep vaginal rugae because of its high viscoelasticity (Caramella et al., 2015; Johal et al., 2016). Additionally, the contamination risk of the remaining drug and irritating friction during administration to infectious mucosa, still remains obstacles for the application of vaginal gel.

Inspired from the distinct properties of aerosol foam and gel, an expansible thermal gelling foam aerosol (ETGFA) was proposed to combine the advantages of foam and gel for drug penetration and carrier retention in vaginal canal, respectively. The ETGFA was administrated as foam aerosol and then thermally transformed into gel at physiological temperature upon contacting vaginal mucosa. Upon being spurted out, the ETGFA foam would expand rapidly, penetrate deeply and distribute directly to the infectious vaginal rugae and remain at action sites by thermal gelation. The superiorities of foam, including spread uniformity, self-administration and patient compliance (alleviating uncomfortable feeling and contamination risk), as well as the superiorities of gel, including mucosal adhesion, prolonged carrier retention and extended drug release, were fully exploited by the newly-designed ETGFA for optimal vaginal drug delivery. Additionally, the presented work also provides some insights to understand the crucial factors influencing the drug delivery efficiency of VDDS dosage forms.

## 2. Materials and methods

### 2.1. Materials

Silver nanoparticles (NTX-300WT) were purchased from Denafu Nanometer Technology Co., Ltd. (Shanghai, China). Propane and butane were obtained from Jiali Daily Chemical Co., Ltd. (Foshan, China). Both poloxamer 407 (WPED612D) and poloxamer 188 (WPOD583B) were purchased from BASF SE (Charlotte, NC). Carbopol (974 P) was obtained from Jiefu Trading Co., Ltd. (Guangzhou, China). Asimi^®^ was purchased from Qinghua Yuanxing Nano-pharmaceutical Co., Ltd. (Shenzhen, China).

*Escherichia coli* (*E. coli*, ATCC 25922), *Staphylococcus aureus* (*S. aureus*, ATCC 25923), *Pseudomonas aeruginosa* (*P. aeruginosa*, ATCC 27853) and *Candida albicans* (*C. albicans*, ATCC 64550) were obtained from American Type Culture Collection (ATCC, Manassas, VA). SD rats were provided by Guangdong Medical Laboratory Animal Center (Foshan, China). Studies were conducted in accordance with guidelines and procedures approved by the Institutional Authority for Laboratory Animal Care and Ethics Committee of Sun Yat-sen University.

### 2.2. Preparation of foam aerosol

The base solution of ETGFA was prepared from thermal sensitive poloxamers, adhesive agents and silver nanoparticles. Specifically, definite amounts of poloxamers and adhesive agents were dispersed into deionized water and were stored at 4 °C for 24 h to acquire homogeneous solution. Combination of 18 ∼ 22 % (w/w) poloxamer 407 and 0 ∼ 5 % (w/w) poloxamer 188 were optimized by gelation temperature (supplemental material). Adhesive agents, including arabic gum, sodium carboxymethyl cellulose (CMC-Na), sodium alginate, carbopol and xanthan gum, were screened according to their adhesive strength (supplemental material). For drug loaded formulation, silver nanoparticles were added to the base solution to obtain a drug concentration of 1% w/w.

Propellants of hydrocarbon (propane/butane = 80/20, v/v) and dimethyl ether were compared based on foam expansion and duration, respectively. To obtain the foam aerosol, the drug-loaded base solution was added into aluminum bottle followed by propellant canning and sealing.

### 2.3. Characterization of foam

Foam appearance, density, expansive extent, duration time and weight loss on drying were characterized, respectively. Foam appearance was photographed with a digital camera (ST76, Samsung, South Korea). For density, the foam aerosol was spurted to a weighted container at 25.0 ± 0.5 °C until the foam fully filled the container. Then the foam was replaced by identical volume of deionized water. Foam density was calculated according to the Equation (1) (Tamarkin et al., 2006):

(1) $$\rho_{\text{f}}=\rho_{\text{w}}•w_{\text{f}}/w_{\text{w}}$$

where *ρ*_w_ is the density of water, *w*_f_ is the weight of foam and *w*_w_ is the weight of water.

To investigate the expansion extent and duration time of the foam, the measuring method using buret was modified from previous literature (Yasumatsu et al., 2014). Briefly, 3 mL base solution of foam aerosol was spurted to a buret with inner diameter of 15 mm at 25.0 ± 0.5 °C. The foam expanded through the canal of buret and shrank back to the bottom after reaching the apex. The expansion volume was calculated by the scale of the buret and the duration time was recorded from the moment when the foam reached maximum volume to its dissipation to the buret bottom. For weight loss on drying (Xiang et al., 2017; Zhu et al., 2017), the ETGFA foam was dried in an oven at 65.0 ± 0.5 °C until the weight of foam remained constant and the weight difference of the foam before and after drying, was calculated according to the Equation (2):

(2) $$\text{weight loss on drying}=\left(w_{\text{1}}-w_{\text{2}}\right)/w_{\text{1}}•\text{1}00\%$$

where *w*_1_ and *w*_2_ are the foam weights before and after drying, respectively.

### 2.4. Gelation temperature and gelation time

To investigate the dilution effect of vaginal fluid on the gelation temperature, the thermal gelation of the ETGFA with and without stimulus vaginal fluid (SVF) dilution, was respectively characterized by a rational rheometer (Kinexus Lab+, Malvern, UK) in the oscillation model from 25 to 40 °C, with frequency (*f*) at 1 Hz and shear strain at 1%. The volume ratio of ETGFA base solution and SVF was 10:3 and the preparation of SVF is shown in the supplemental material (Owen & Katz, 1999; Chang et al., 2002).

In addition, the gelation time from foam to gel was determined by the same rheometer at 37.0 ± 0.5 °C, with *f* at 1 Hz and shear strain at 1%.

### 2.5. Adhesive strength of the gels

**Figure 1.**
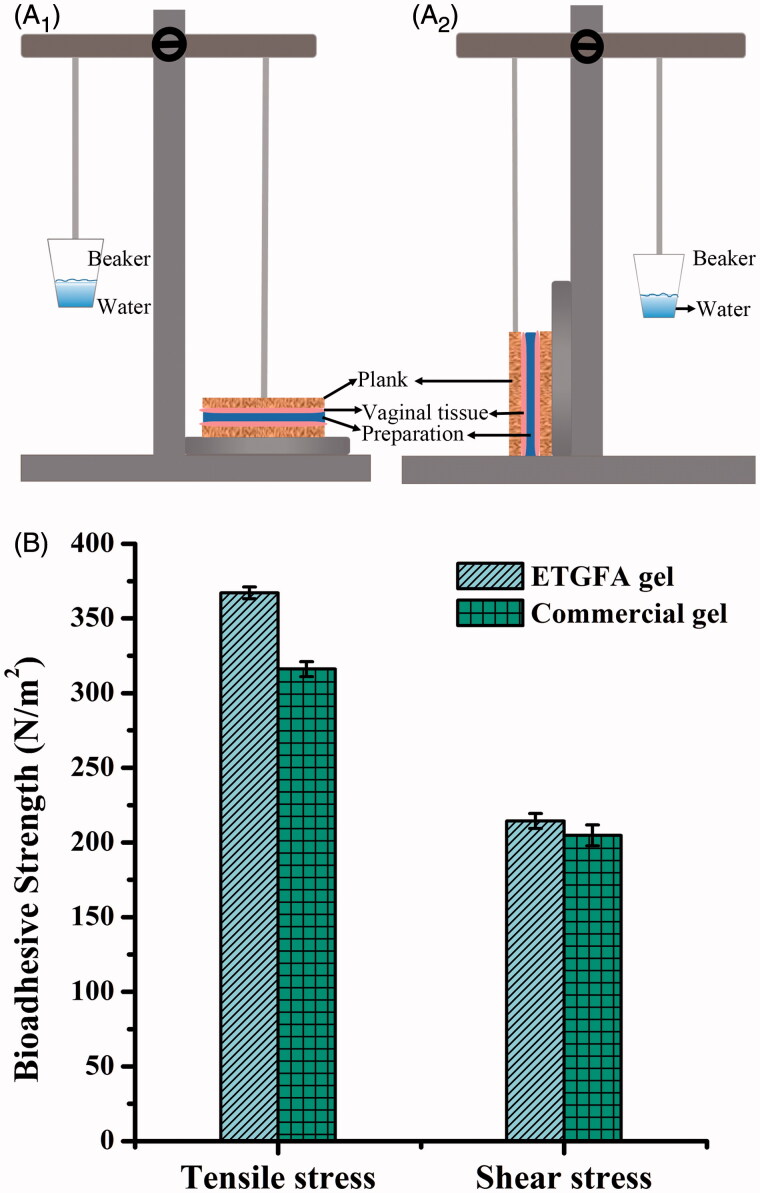
Schematic illustration of the devices and the results of adhesive strength evaluation (*n* = 3). (A) the schematic illustration of the devices used for evaluation of adhesive strength, A_1_ for tensile stress and A_2_ shear stress; (B) adhesive strength results of the ETGFA gel and the commercial vaginal gel Asimi^®^.

The adhesive strength of the ETGFA gel and the commercial vaginal gel Asimi^®^ was evaluated by the devices (Figure 1(A_1_, A_2_)) modified from Vermani et al.’s research (Vermani et al., 2002) at 37.0 ± 0.5 °C. To simulate the dilution effect of vaginal fluid on the adhesive strength, the ETGFA was spurted into SVF at a volume ratio of 10:3 to obtain pretreated ETGFA gel. The planks of the devices were wrapped with isolated vaginal tissues pretreated with SVF.

**Figure 2.**
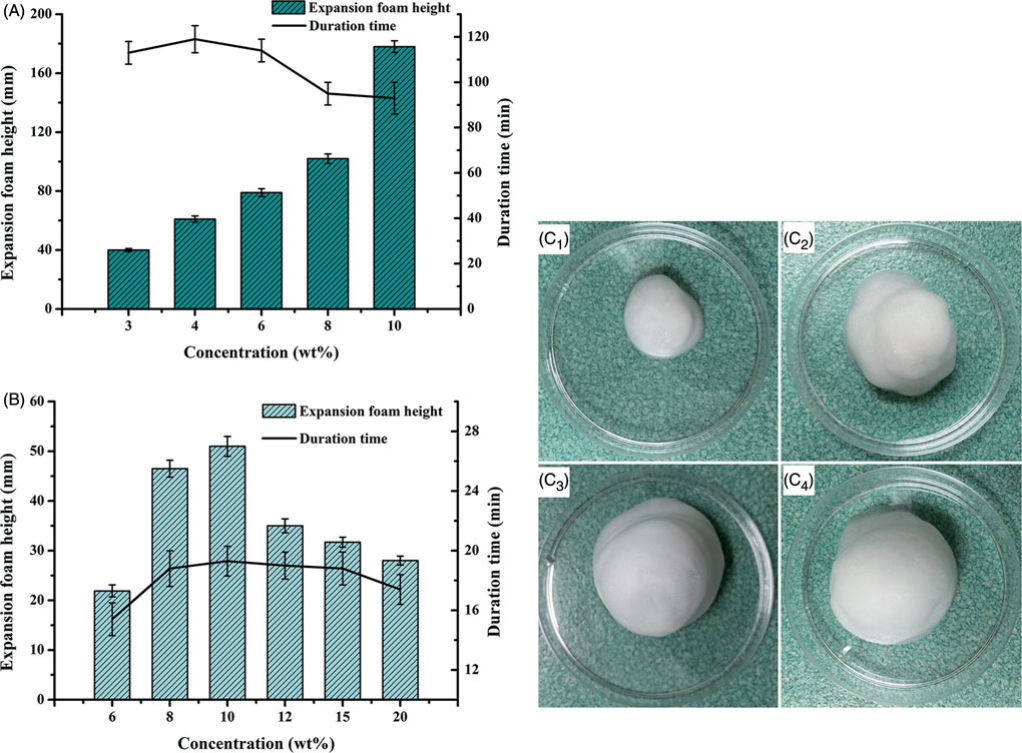
Comparison of expansion height and duration time of foam (*n* = 3) propelled by (A) hydrocarbon (propane/butane = 80/20, v/v) ranging from 3 to 10%, w/w as well as (B) dimethyl ether, ranging from 6 to 20%, w/w; and the foam appearances from expansion to collapse (25.0 ± 0.5 °C) at 0 min (C_1_), 2 min (C_2_), 10 min (C_3_) and 60 min (C_4_), respectively.

0.1 g pretreated ETGFA gel was uniformly spread on the surface of the bottom plank. The top plank contacted tightly with the bottom plank, remaining in a balance state. Definite amount of water was dropwise added to a tared beaker fixed on the opposite side of the balance. Once the two planks were separated, the weight of water was recorded as *w*_1_ and the tensile stress *σ* was calculated according to the Equation (3):

(3) $$\sigma \;=\left(w_{1}-w_{0}\right)/s$$

where *w*_0_ is the weight of top (Figure 1(A_1_)) or left plank (Figure 1(A_2_)), and *s* is the contacting area between the formulation and the isolated vaginal tissues.

For shear stress, the two planks were rearranged from top-bottom to left-right position with the other steps the same as that in the measurement of tensile stress. The weight of water was recorded as *w*_1_ once the left and right planks were separated and the shear stress *τ* was calculated the same as the tensile stress according to the Equation (3).

### 2.6. Viscosity of the base solution and ETGFA gel

Viscosity of both the base solution and the gel of ETGFA was evaluated by a rational rheometer (Kinexus Lab+, Malvern, UK). The sample was loaded to bottom plate and then the top plate was lowered to a predetermined position. Viscosity measurement post thermal equilibrium was conducted from 0.1 ∼ 100 s^−1^ at 25.0 ± 0.5 °C for base solution and 37.0 ± 0.5 °C for gel, respectively.

### 2.7. *In vitro* release test

*In vitro* drug release test of the ETGFA was carried out using a membrane-free dissolution method. 1.5 g drug-loaded ETGFA foam was spurted to a vial with 0.45 mL SVF medium at 37.0 ± 0.5 °C and thermally transformed into gel within 2 min. At predetermined time intervals, the medium sample was withdrawn completely and replaced by equivalent fresh medium. The collected sample was filtered through 0.45 μm filter prior to drug quantification by atomic absorption spectrophotometry (SOLAAR S4, Thermo Fisher, Waltham, MA) with following parameters: detection wavelength at 328.1 nm; lamp current at 5.0 mA; airflow at 6.0 L/min; acetylene flow at 1.1 L/min; slit width at 0.5 nm and burner height at 7.0 mm. *In vitro* drug release behavior of the commercial vaginal gel Asimi^®^ was tested using the same method.

### 2.8. Minimum inhibitory concentration (MIC) of silver nanoparticle on *P. aeruginas, S. aureus, E. coli* and *C. albicans*

Silver nanoparticle-loaded ETGFA in the diluted concentrations of 200, 100, 50, 25, 12.5, 6.25, 3.125 and 0 (positive control without drug) μg/mL, respectively were added to sterile tubes numbered from No. 1 to No. 8. Subsequently, 200 μL bacteria suspensions of *P. aeruginas, S. aureus, E. coli* or *C. albicans* equivalent to 0.50 McFarland standard, were added to achieve a bacteria concentration of 1 × 10^7^ cfu/mL, respectively. Another tube (No. 9) containing diluted silver nanoparticle-loaded ETGFA (3.125 μg/mL) but no bacteria suspension, was set as a negative control. The tubes were sealed and were kept for shaking for 24 h at 37.0 ± 0.5 °C, 120 rpm. For colony observation, samples were transferred from the tubes to the culture dishes which were incubated at 37.0 ± 0.5 °C post to addition of 20 mL agar. After 72 h, the minimum concentration of silver nanoparticle without any colony growth in the culture dish was regarded as the MIC of each tested bacterium.

As the control group, the MIC of silver nanoparticle solution on the four bacterias was determined by the same method.

### 2.9. Bacterial growth curve of *P. aeruginas, S. aureus, E. coli* and *C. albicans*

For the bacterial growth curve, 200 μL silver nanoparticle-loaded ETGFA at drug concentrations of 0, 5, 10, 50 and 100 μg/mL, were mixed with 2 μL bacteria suspension of *P. aeruginas, S. aureus, E. coli* or *C. albicans* equivalent to 0.5 McFarland standard, respectively. The mixture was incubated at 37.0 ± 0.5 °C. After 1, 2, 4, 8, 10 and 12 h, optical density (OD) values of the bacteria-ETGFA mixture were measured with a spectrophotometer (ELX800, BioTek, Winooski, VT). Bacteria growth curve was plotted with OD value versus time.

### 2.10. *In vivo* irritation test

Sixteen female SD rats (200 ± 20 g) were randomly divided into four groups: group A for saline, group B for blank ETGFA, group C for silver nanoparticle-loaded ETGFA and group D for silver nanoparticle solution (group C and D equivalent to a dosage of 0.5 mg silver nanoparticles/kg). After vaginal administration of equivalent volume of samples daily for a consecutive week, rats were sacrificed for pathological assessment.

For hematoxylin-eosin staining (HE staining), the specimens were withdrawn from ovary, uterus and vagina. After fixation by 10% formalin, the specimens were dehydrated for 10 h and paraffined. The paraffined specimens were sectioned into 3 µm thick slices and baked at 65.0 ± 0.5 °C for 30 min. After preservation at 60.0 ± 0.5 °C for 12 h, the specimens were dewaxed and treated with the following agents: gradient ethanol (100, 95 and 85%, v/v) hydration for 5 min, respectively, hematoxylin for 10 min, saline for 10 min, 95% (v/v) ethanol for 2 min, 0.5% (w/v) eosin for 2 min and gradient ethanol (100, 95 and 85%, v/v) dehydration for 5 min, respectively. After mounted in the neutral resins, the specimens were observed and photographed by the microscope equipped with a digital camera.

Irritation scores were rated for epithelial tissue swelling, leucocyte infiltration, vascular congestion and edema at vagina, ovary and uterus. Irritation of the treated groups was demonstrated by comprehensive irritation index (Dover et al., 2007; Bachhav & Patravale, 2009; Li et al., 2016) (shown in supplemental material).

### 2.11. Statistical analysis

Statistical analysis among multiple groups was analyzed by one-way ANOVA using Tukey test in the SPSS software (SPSS version 19.0; SPSS Inc., Chicago, IL).

## 3. Results

### 3.1. Optimization and characterization of the ETGFA foam

The ETGFA formulation were optimized by selecting the compositions and concentrations of the thermal matrices, adhesive agents and propellants according to the results of gelation temperature (Table S1, S2), adhesive strength (Figure S1) and foam properties, respectively. The optimal base solution consisted of poloxamer 407, poloxamer 188, carbopol, silver nanoparticle, water in a weight ratio of 21:6.5:0.2:0.01:72.3.

The gelation temperatures of the optimal base solution were recorded at 28.8 ± 0.3 °C without SVF and 35.7 ± 0.3 °C with dilution of SVF (Table S1, S2). The formation of foam is dependent on the propellant, which also has an important influence on the foam properties, such as expansion extent and duration time. Good expansion extent of foam is desirable for drug penetration deep into the infectious sites. Also, duration time should be long enough to allow thermal gelation of the ETGFA before foam collapse to effectively avoid foam liquefaction and drug leakage.

In present study, the commonly used propellants, liquefied hydrocarbon (propane/butane = 80/20, v/v) and dimethyl ether (Caballero et al., 2008; Kakami et al., 2011; Azizi et al., 2014), were applied and the effects of their concentrations on the foam expansion and duration were compared, respectively. The hydrocarbon was selected as the propellant due to its capacity to generate the foam with higher expansion height and longer duration time than dimethyl ether (Figure 2(A, B)). Additionally, the required concentration of hydrocarbon to achieve sufficient foam expansion and duration was much lower than that of dimethyl ether. The foam generated by 4 wt% hydrocarbon possessed superior expansion height (64.8 ± 2.5 mm) and duration time (123.2 ± 5.6 min) than that of the optimal foam propelled by dimethyl ether in the concentration range of 6 ∼ 20 wt% (maximum expansion height of 54.3 ± 1.1 mm and maximum duration time of 18.7 ± 1.6 min, respectively). The foam propelled by hydrocarbon in the concentration range of 3 ∼ 10 wt%, reached maximum values of 169.3 ± 3.7 mm for expansion height and 123.2 ± 5.6 min for duration time, respectively (Figure 2(A)). Hydrocarbon concentration was chosen at 4 wt% to acquire desirable foam expansion and duration and to reduce potential tissue irritation. Therefore, the optimized ETGFA formulation consisted of 96 wt% base solution and 4 wt% hydrocarbon.

Foam properties of the optimized ETGFA formulation, including foam appearance, density and weight loss on drying, have great influence on the vaginal delivery efficacy of the silver nanoparticles and thus were comprehensively characterized. Specifically, the ETGFA foam was in pale white color (Figure 2(C)). The foam was dense and in a relative small volume (Figure 2(C_1_)) upon being spurted out from the sealed container and then expanded to a much larger size with puff texture (Figure 2(C_2_,C_3_)), reaching the maximum expansion state within 10 min. The foam was light with a density of approximately 0.10 g/mL at its maximum volume. The foam retained the maximum expansion up to 60 min, followed by collapsing process with gradual liquefaction (Figure 2(C_4_)). Loss on drying of the foam was rather high, which was up to 82.6% of the weight of the spurted formulation.

**Figure 3.**
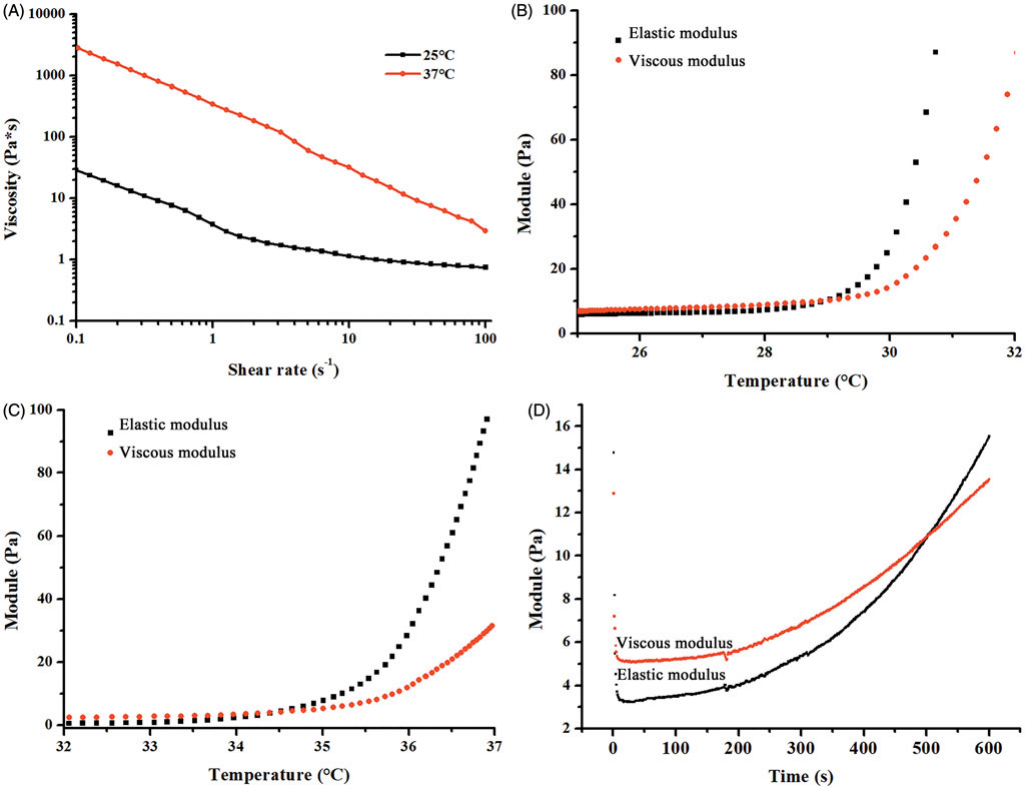
Rheological properties of the ETGFA. (A) viscosity of the ETGFA at 25.0 ± 0.5 and 37.0 ± 0.5 °C, respectively; (B) viscoelasticity of the ETGFA from 25 to 32 °C; (C) viscoelasticity of the ETGFA diluted with stimulus vaginal fluid from 32 to 37 °C and (D) gelation time of the ETGFA diluted with stimulus vaginal fluid from foam to gel at 37.0 ± 0.5 °C.

**Figure 4.**
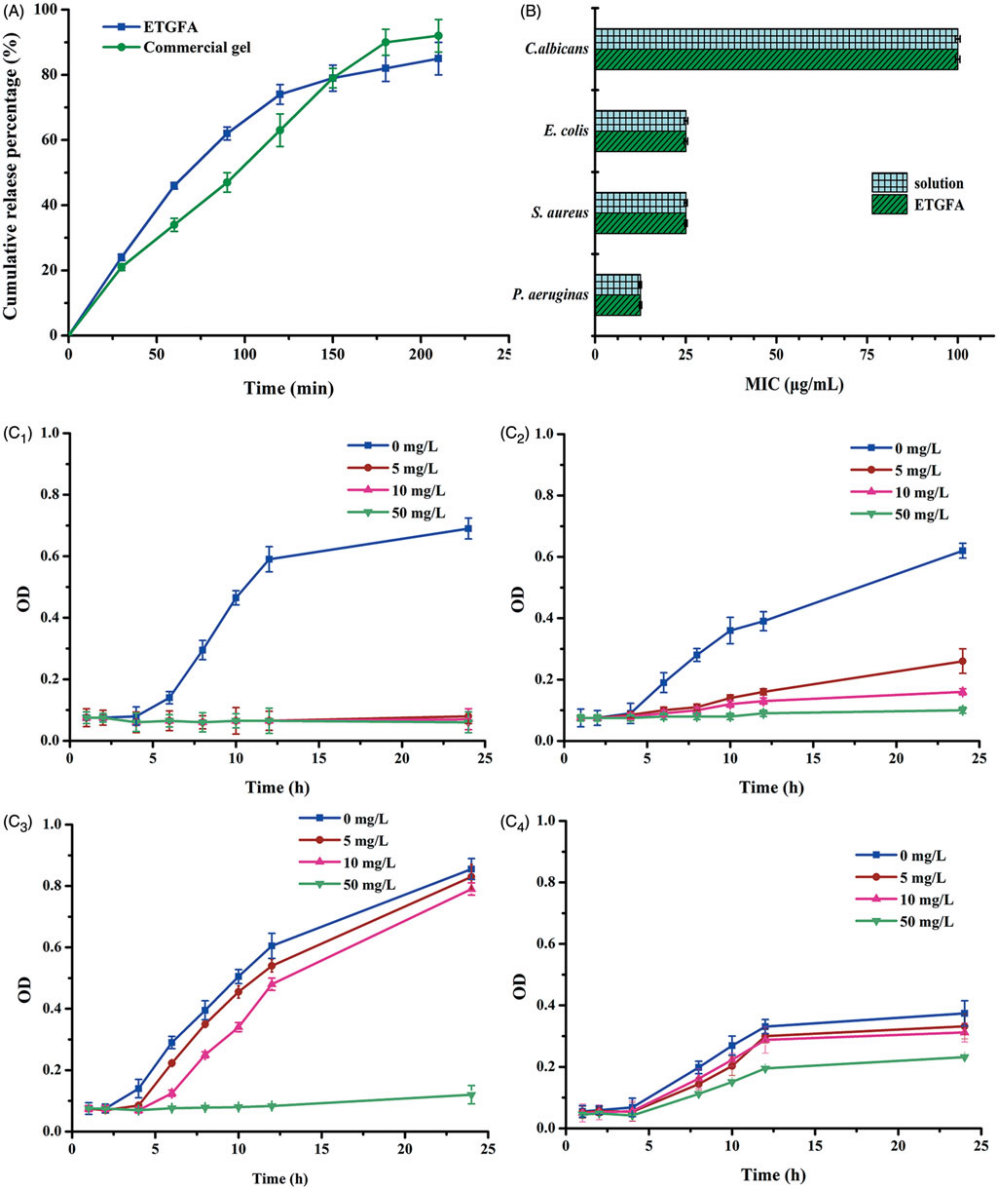
*In vitro* drug release and antimicrobial effect. (A) *in vitro* drug release from the ETGFA and the Asimi^®^ determined by membrane-free method at 37.0 ± 0.5 °C (*n* = 3); (B) MICs of silver nanoparticle solution and the drug-loaded ETGFA on *P. aeruginas*, *S. aureus*, *E. coli* and *C. albicans* (*n* = 6) and (C) bacteria growth curves treated with ETGFA containing different silver nanoparticle concentrations (*n* = 6), (C_1_) *P. aeruginas*; (C_2_) *S. aureus*; (C_3_) *E. coli* and (C_4_) *C. albicans*.

### 3.2. Viscosity, gelation temperature and gelation time of the ETGFA

#### 3.2.1. Viscosity of the base solution and gel

Viscosity of the base solution and gel was characterized to assess the rheological performance of the ETGFA during foam spurting and gel retention in the vaginal canal. Appropriate viscosity was required to reduce the friction during spurting process. The base solution showed shear thinning property at 25.0 ± 0.5 °C and its viscosity declined from 28.43 Pa·s at 0.1 s^−1^ to 0.73 Pa·s at 100 s^−1^ (Figure 3(A)). During spurting, the vigorous shearing of the base solution led to dramatic reduction of the viscosity, which would markedly reduce the friction force for the flow of the base solution, which facilitates the out-spurting process and improves the administrated feasibility of the ETGFA for vaginal application (Hari et al., 2015).

The viscosity of ETGFA gel showed similar shear thinning property at 37.0 ± 0.5 °C compared to that of ETGFA base solution at 25.0 ± 0.5 °C with the same shear rate, but its value increased by two orders of magnitude. The dramatical increase in viscosity was induced by the thermal gelation from solution at 25 °C to gel at 37 °C. The high viscosity of gel is in favor of adhesion of drug carrier to vaginal mucosa.

#### 3.2.2. Gelation temperature and gelation time of the ETGFA

The gelation temperature was indicated by the viscoelasticity variation with increasing temperature from 25 to 37 °C. The viscoelasticity is composed of viscous and elastic modulus, respectively that demonstrates the liquid- and solid-like properties of the tested sample. The relative higher value of viscous modulus than that of elastic modulus indicates solution state of the sample while higher elastic modulus indicates gel state and equal values of both moduli indicate solution-gel transition of the sample. The gelation temperature of pure ETGFA was recorded 29.2 °C (Figure 3(B)), indicating that undesirable gelation could to be avoided before administration during the preservation and storage of the formulation at room temperature (approximately 25 °C). The gelation temperature of the ETGFA diluted with SVF rose to 34.5 °C (Figure 3(C)), which could be easily achieved at the physiological condition (approximately 37 °C) with consideration of the dilution effect of vaginal fluid when the ETGFA was spurted to vaginal canal.

The gelation time of the ETGFA at 37.0 ± 0.5 °C was 8.7 min (Figure 3(D)), which allowed the foam to expand and to reach its approximately maximum volume. Importantly, the gelation time was much shorter than the foam duration time (123.2 min, Figure 2(A)), indicating that the complete transformation of ETGFA from foam to gel before the foam collapsing could be achieved by avoiding undesirable drug leakage.

### 3.3. Adhesive strength

The ETGFA foam thermally transformed into *in situ* gel after contacting vaginal mucosa at physiological temperature. Adhesive strength of the *in situ* gel, including tensile stress and shear stress, were evaluated to investigate its mucosal adhesion. Adhesive agents were added to improve mucosal adhesion and five adhesive agents, including arabic gum, sodium carboxymethyl cellulose, sodium alginate, carbopol and xanthan gum, were compared with herein. Carbopol was selected as the optimal adhesive agent for its superior adhesive strength (Figure S1) and lower viscosity. Subsequently, the adhesive strength of the ETGFA gel containing 0.2 wt% carbopol was determined and compared with that of the commercial gel Asimi^®^ (Figure 1(B)). Tensile stress of the ETGFA gel was recorded 367.12 ± 3.04 N/m^2^, which was higher than that of Asimi^®^ (316.52 ± 6.27 N/m^2^). Shear stress of the ETGFA gel was 214.35 ± 3.62 N/m^2^, which was also higher than that of Asimi^®^ (204.73 ± 7.13 N/m^2^). It suggested that the ETGFA gel possessed better adhesive strength than that of the commercial vaginal gel Asimi^®^.

### *3.4. In vitro* drug release

Drug release behaviors of the ETGFA and Asimi^®^ were compared using membrane-free method at 37.0 ± 0.5 °C. Both ETGFA and Asimi^®^ demonstrated an extended drug release over 4 h (Jesse, 2016; Nahler, 2016) (Figure 4(A)). The initial drug release from the ETGAF within the first 1 h rapidly provided adequate drug concentration to achieve the effective treatment level, whereas prolonged drug release was extended during later stage to acquire durable antimicrobial effects. The *in vitro* drug release behavior of the ETGFA was comparable to that of Asimi^®^.

### 3.5. Antimicrobial efficacy of the ETGFA

#### 3.5.1. MICs of silver nanoparticle solution and the drug-loaded ETGFA on *P. aeruginas*, *S. aureus*, *E. coli* and *C. Albicans*

MIC test was conducted to evaluate the antimicrobial efficacy of silver nanoparticle-loaded ETGFA. The MIC results demonstrated that the general vaginal floras, including *P*. *aeruginas*, *S. aureus*, *E. coli* and *C. albicans*, had different sensitivities to the silver nanoparticle incorporated in the ETGFA (Figure 4(B)). The viability of both *E. colis* and *S. aureus* was inhibited at 25 μg/mL. *P. aeruginasa* was more sensitive as its viability was inhibited at a lower concentration of 12.5 μg/mL. On the contrary, *C. albicans* was more resistant to the silver nanoparticle incorporated into the ETGFA than the other bacteria with a higher MIC of 100 μg/mL. The MICs of the silver nanoparticle-loaded ETGFA were equivalent to that of free silver nanoparticles in the solution on the same tested bacteria (Figure 4(B), detailed results are shown in Table S3, S4), indicating that the incorporation of sliver nanoparticles into the ETGFA did not attenuate the *in vitro* antimicrobial efficacy of silver nanoparticles.

#### 3.5.2. Bacteria growth curves of *P. aeruginas, S. aureus, E. coli* and *C. albicans*

Bacteria growth curves (Figure 4(C)) were intended to explore the detailed information about the antimicrobial effects of silver nanoparticle-loaded ETGFA. The lag phases of four growth curves, including *P. aeruginas*, *S. aureus*, *E. coli* and *C. albicans*, were extended by the ETGFA containing effective concentrations of silver nanoparticles, compared to that of the same bacteria without drug treatment (the blank ETGFA containing no silver nanoparticle). The antimicrobial effect of silver nanoparticle was dose-dependent as it was enhanced with the increase in silver nanoparticle concentration (Figure 4(C)). When the concentration of silver nanoparticle reached 5.0 μg/mL, *P. aerugina* was completely inhibited (Figure 4(C_1_)). For both *S. aureus* and *E. coli*, only partial inhibitory efficacy was observed at silver nanoparticle concentration of 5.0 μg/mL, and complete inhibition was achieved when the drug concentration reached 50.0 μg/mL (Figure 4(C_2_,C_3_)). *C. albicans* showed the greatest resistance because it required a higher drug concentration over 50.0 μg/mL to obtain effective antimicrobial results (Figure 4(C_4_)). The results of bacterial growth curves were in accordance with the MIC results of corresponding bacteria.

### *3.6. In vivo* irritation test

**Figure 5.**
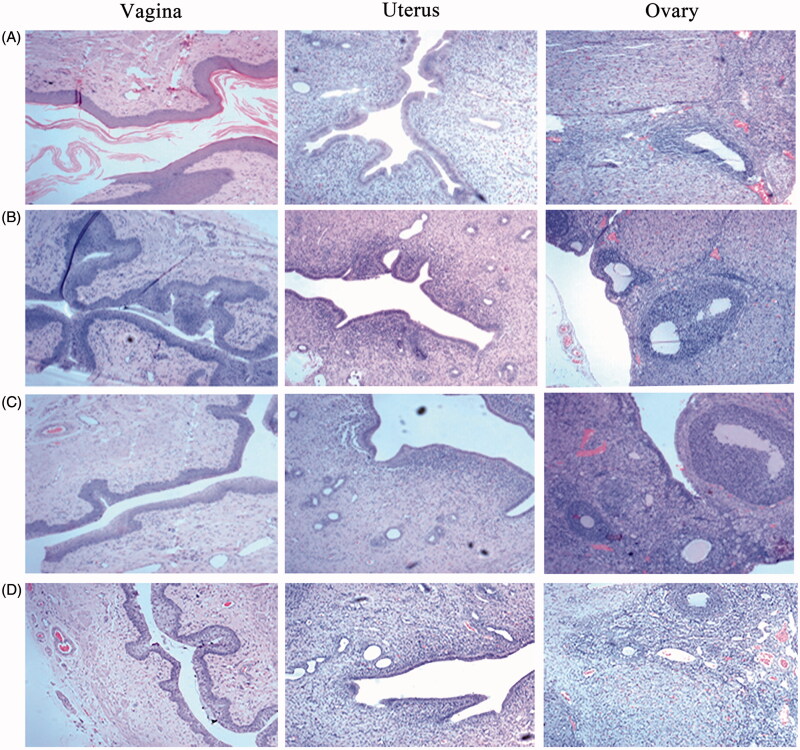
Pathological sections of vaginal, uterine and ovary tissues from rats. (HE staining, ×400). (A) saline; (B) blank ETGFA; (C) silver nanoparticle-loaded ETGFA and (D) silver nanoparticle solution.

Histopathological test provided a visual inspection of the tissues from vagina, uterus and ovary after vaginal administration of saline, blank ETGFA, silver nanoparticle-loaded ETGFA and silver nanoparticle solution, respectively. For control group treated with saline (Figure 5(A)), vaginal epithelial tissue and uterine endometrium were intact. Oocytes were clearly observed in the ovary. No inflammatory cell infiltration, vascular congestion or edema was observed in the tested tissues. For blank ETGFA (Figure 5(B)), histopathological inspection was the same as that of control group except for hyperkeratosis of vaginal epithelial tissue and slight uterine eosinophilic infiltration (<25/high power field, HPF) (Folpe et al., 2005). For the silver nanoparticle-loaded ETGFA (Figure 5(C)), vaginal epithelial hyperkeratosis and slight uterine eosinophilic infiltration (<25/HPF) were also observed. In addition, local infiltration of lymphocytes (5 ∼ 10 %) emerged in the lamina propria of endometrium. The tissue irritation of silver nanoparticle solution was the most serious among the tested groups. Comprehensive irritation index, defined as the difference between irritation scores of the treated group and that of the control group of saline, was calculated for further evaluation of the tissue irritation (the calculate method and detailed results are shown in Tables S5 and S6). The comprehensive irritation index of silver nanoparticle-loaded ETGFA was 2.66 while that of silver nanoparticles was 3.25 (Table S7).

## 4. Discussion

Commercial foam and gel are the commonly used dosage forms for the treatment of vaginal infection, but suffers from drug leakage and incomplete antimicrobial effects (Johal et al., 2016; Rencber et al., 2017). For foam, the drug retention was limited due to the removal of drug carrier by vaginal secretion and self-cleaning. For gel, it is unable to penetrate to the infection sites in deep vaginal rugae.

**Figure 6.**
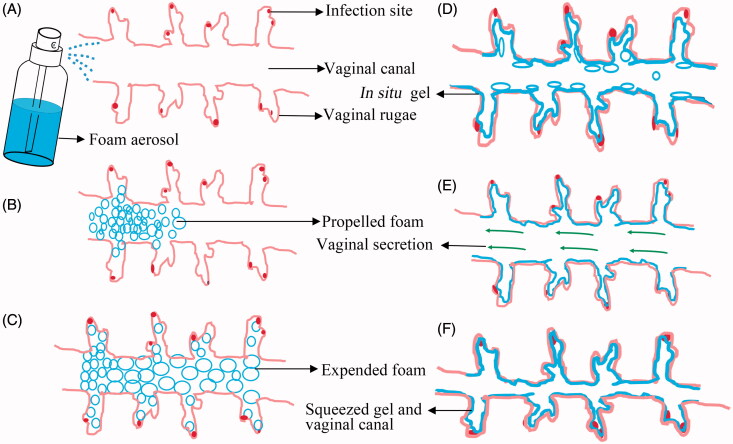
The schematic illustration of the expansible thermal gelling foam aerosol during foam expansion, thermal gelation and gel retention. (A) the ETGFA is administrated as foam aerosol; (B) the foam aerosol is spurted to the vaginal canal by propellant; (C) the foam expands and penetrates to the infectious sites in deep vaginal rugae; (D) the foam thermally transforms into gel to cover the infectious sites on the vaginal mucosa and the gel resists to the self-cleaning during (E) vaginal secretion and (F) vaginal contraction motion.

Promisingly, the ETGFA may be a candidate dosage form for the treatment of vaginal infection. The ETGFA was designed as a thermal gelling drug carrier with foam-gel phase transition to address the issue of short drug retention for foam and that of indirect drug delivery for gel (Figure 6). Specifically, the ETGFA has superiorities, including foam expansion to fully covering the infectious sites, improved mucosal adhesion and prolonged drug retention due to the gelation process.

### 4.1. Foam properties

The favorable foam properties, such as low density, superior expansion and long duration, were achieved by utilizing optimal propellant. Propellant was an essential component for the formation, expansion and duration of foam. Liquefied hydrocarbon and dimethyl ether were reported to be promising propellant candidates because of their good safety and propelling capacity (Arzhavitina & Steckel, 2010). Hydrocarbon (propane/butane = 80/20, v/v) was selected as the propellant for the ETGFA after screening because the foam generated by it exhibited higher expansive height and longer duration time than that by dimethyl ester (Figure 2(A,B)).

In this study, the foam expansive height was recorded up to 64.8 mm, which suggested favorable foam swelling after administration. Additionally, the expansive foam had low density of 0.10 g/mL. Great expansion and low density make the foam spread directly and deeply into the twisted vaginal rugae (Figure 6(B,C)). The infectious sites embedded in the vaginal rugae could also be treated with effective drug concentrations instead of insufficient contact with antimicrobial drugs. The direct exposure of bacteria to the antimicrobial drug would effectively inhibit advance and recurrence of vaginal infection. In addition, expansion of definite amount of foam could reduce the film thickness of the foam bubbles so that the foam had larger contact area with vaginal mucosa, which would enhance the local absorption of the antimicrobial drug (Torchilin, 2007). The foam was homogeneous and soft (Figure 2(C)), indicating superior drug uniformity and comfortable feeling during administration.

The prolonged duration of the foam could be explained by two properties of hydrocarbon: lower solubility in the base solution and lower surface tension. Lower solubility of the hydrocarbon in aqueous domain could retard the foam collapsing process by slowing down the gas diffusion from small to large foam bubbles (Augsburger & Shangraw, 1968), while lower surface tension contributes to maintaining foam in a thermodynamically stable state. Consequently, the propellant of hydrocarbon contributed to the formation of stable foam with a longer duration of 123.2 min, which guaranteed sufficient time for complete phase transition from foam to *in situ* gel (Arzhavitina & Steckel, 2010).

Also, the results showed that the duration time of the foam was much longer than the gelation time of 8.7 min (Figure 3(D)), which prevented undesired foam liquefaction and collapsing before thermal gelation. The gelation time was dependent on the lag for thermal conduction between the ETGFA and ambient environment. The foam expansion after vaginal administration created huge contact area between the foam and vaginal mucosa, which would facilitate the thermal conduction, consequently inducing faster foam-gel transition. This was the key point to avoid drug leakage and prolonged drug retention.

### 4.2. Thermal gelation of the ETGFA

A variety of dosage forms for the treatment of vaginal infection, especially the liquid dosage forms, were limited by short retention time (Valenta, 2005; Kieweg & Katz, 2006). The administrated formulations were easy to be washed from the vaginal canal and thus fail to achieve desirable therapeutic effects owing to the insufficient contacting time. The ETGFA in presented study possessed a thermal transition from foam during administration (Figure 6(A)) to gel at action sites for prolonged drug retention (Figure 6(D)).

Several tri-block copolymers could be utilized as thermal-induced gelation matrix (Woodcock et al., 2010; O’Lenick et al., 2011; Henn et al., 2014). Poloxamer, a tri-block copolymer, played a key role in the thermal gelation (Cao et al., 2010), which was driven by the piling and entanglement of its blocks of hydrophilic polyethylene oxide and hydrophobic polypropylene oxide. Generally, the gelation process was both concentration and temperature dependent (Djekic et al., 2015; Calderas et al., 2016). When poloxamer reached the critic micelle concentration, micelles formed gradually and contacted mutually. The compact micelle stacks shaped the structure of solid spherules and triggered the solution-gel transition. The gelation process was also dependent on the temperature variation. Gel was formed by the aggregation of poloxamer molecules through dehydration effect when the temperature exceeded the critic micelle point.

In present design of the ETGFA, the concentrations of poloxamers were adjusted to trigger the thermal gelation at physiological temperature and avoid undesirable gelation at room temperature. Gelation is difficult to achieve at vaginal canal when the poloxamer concentration was too low for gelation at physiological temperature, whereas high poloxamer concentration would inevitably induce the gelation process at room temperature, resulting in obstacles in transportation, storage and administration of ETGFA (Cabana et al., 1997; Bohorquez et al., 1999). In this formulation, an optimized combination of 21 wt% poloxamer 407 and 6.5 wt% poloxamer 188 was developed to modulate the gelation temperature. The gelation temperature of pure ETGFA was 29.2 °C, which prevented undesirable solution-gel phase transition before administration. After administration, the ETGFA would be diluted by the vaginal fluid and the gelation temperature increased to 34.5 °C, which could assure the thermal gelation at physiological vaginal environment with consideration of the dilution effect of vaginal fluid. The optimized gelation temperature guaranteed a proper foam-gel transition with improved administration compliance and prolonged drug retention.

### 4.3. Rheological properties of the ETGFA

The ETGFA remained solution before administration, whose low viscosity and shear thinning property allowed easy spurting of the aerosol, improving the administration convenience and causing less discomfort to the infectious sites during administration. The non-linear relationship between the viscosity and shear rate revealed the non-Newtonian pseudo-plastic fluid property of the base solution. As shear rate increased, the viscosity of the base solution decreased. Vigorous shear was imposed to the base solution when the valve opened and the propellant vaporized. The viscosity of the base solution was dramatically reduced so that less energy was required for the flow of base solution and it would be easier to be spurted out to the vaginal canal. Compared with the conventional vaginal gel applied with fingers or tools, the foam was spurted to the vaginal canal and spontaneously expanded into the deep infectious sites, reducing discomfort feeling. Patient compliance was improved by reducing friction and irritation during the vaginal administration of conventional gel.

The viscoelasticity property was also important for the ETGFA application. Viscoelasticity includes viscous modulus (indicating liquid-like property) and elastic modulus (indicating solid-like property). The viscous modulus was related to the softness of foam which could reduce irritation to the infectious sites. The elastic modulus demonstrated the resilient capacity under the exterior force from mucosal rugae during vaginal motion. The ETGFA with intensive elastic modulus possessed adequate stretching and adhesion capacity.

Both the elastic and the viscous modulus, respectively increased with rise in the temperature. The increase in both modulus was dramatic and the elastic modulus overpassed the viscous modulus at the gelation temperature, owing to the thermal-induced gelation process. The newly formed gel could not only improve the inter-molecules interaction between the poloxamer molecules and glycoproteins in the mucus, but also could inhibit the vaporization of the remaining liquid film to prevent foam collapse. The elastic modulus ascended as the temperature rose and the more solid property was obtained to resist the mechanic damage from vaginal canal (Figure 6(E,F)).

For conventional foam, the elastic modulus significantly declined as intermolecular distance was enlarged and intermolecular interaction was weakened due to increase in the temperature (Kealy et al., 2008). The reduced elasticity resulted in the issues of instability and short drug retention. Additionally, the liquid film on foam surface evaporated quickly and facilitated the foam collapsing at higher temperature (Weaire et al., 2001). Decreasing elastic modulus made the foam vulnerable to mechanic damage from the vaginal motion, leading to the destruction and loss of the drug carrier from the vaginal canal. Promisingly, the issues were addressed by the addition of adhesive carbopol and foam-gel transition in presented study. After administration, the foam stuck to the mucosa by the interaction of carbopol polymers and glycoproteins in mucus. The elongation of retention time was also attributed to the phase transition of the ETGFA from foam to gel. The thermal gelation induced by temperature shift contributed not only to the direct drug distribution to the infectious sites by foam expansion but also to the prolonged drug retention by the *in situ* gel with intensive elasticity, to achieve desirable *in vivo* antimicrobial effect.

### 4.4. Adhesive strength of the ETGFA gel

The adhesive strength of ETGFA gel was assessed in order to investigate its retention at the infectious sites. Adhesive strength indicated the capacity of gel to stick to the tissues (Gupta et al., 2016). Vaginal mucosa secretes mucus containing adhesive glycoproteins. The polymers in the gel could swell, penetrate into the adhesive mucus and intercross with glycoproteins in the mucus by hydrogen bond, electrostatic attraction and hydrophobic force. Carbopol was selected as adhesive agent for its capacity of physically intercrossing with mucosal glycoproteins and forming hydrogen bond (Bonacucina et al., 2006; Jones et al., 2009; Surassmo et al., 2015).

In present study, carbopol contributed to the strong adhesiveness of the thermal transformed gel, which was better than that of Asimi^®^ and was intended to offer longer retention time at infectious sites. It could overcome the limitation of drug removal and consequently avoid multiple and frequent administrations.

### *4.5. In vitro* drug release and antimicrobial efficacy

The *in vitro* drug release behavior indicated no significant difference between the cumulative drug release from ETGFA and that from Asimi^®^ at each time interval. Asimi^®^ is a commercially available silver nanoparticles gel to treat vaginal disease, which is effective for the treatment of mild, moderate and severe vaginitis and cervical erosion induced by microbial infection. Patients only have to administrate the Asimi^®^ gel for once a day clinically.

The *in vitro* drug release profiles of ETGFA and the Asimi^®^ gel was similar, suggesting comparable *in vivo* behavior and therapeutic efficacy. However, some differences between two drug release curves were observed. Slightly higher initial drug release from the ETGFA than that from the Asimi^®^ gel was determined. This could be explained by the lagging gelation process, which was mainly associated with the time lag for thermal conduction between the ETGFA and dissolution medium. Before complete transformation into gel, foam was the dominant form of the ETGFA, which possessed rapid drug release rate due to its loose structure and large contact area with dissolution medium. The initial burst release provided effective drug concentrations above the MICs of the general vaginal flora (Silva et al., 2017) and achieved desirable antimicrobial effects. The drug concentration in the ETGFA (approximately 100 μg/mL) was equal to the MIC of the most resistant vaginal bacteria *C. albicans.* As the content of vaginal fluid is much smaller than the volume of administrated ETGFA, the drug concentration in both ETGFA and vaginal fluid was supposed to be identical after a short time because of the initial burst release. The initial drug concentration in the vaginal fluid was comparable to the MIC of the most resistant vaginal bacteria. The subsequent extended release achieved by incorporation of the silver nanoparticles into ETGFA gel, would elongate the drug retention time. The local drug concentration remained stable and comparable to the MICs of general vaginal flora, avoiding undesirable overdose and accumulation of the antimicrobial drug at the infectious sites, which was the main cause of the drug resistant and tissue irritation.

To determine the influence of the silver nanoparticles incorporation into ETGFA on the therapeutic effects, bacterial inhibitory efficacy of both silver nanoparticle-loaded ETGFA and the silver nanoparticle solution were compared by their MICs on *P. aeruginas, S. aureus, E. coli* and *C. albicans*. MIC results clearly demonstrated that drug incorporation did not affect the antimicrobial efficacy compared with silver nanoparticle solution (Figure 4(B), detailed results is shown in Table S3, S4). The antimicrobial efficacy was achieved by the release of silver nanoparticles and their penetration into the bacterial cells, which tended to attack the respiratory chain of the treated cells (Rai et al., 2009). The ETGFA provided effective concentrations comparable to the MICs of silver nanoparticle to the four pathogenic vaginal floras for comprehensive antimicrobial activity.

Antimicrobial effect was also explored by comparing the bacterial growth curves of the four pathogenic vaginal floras with increasing drug concentrations. The results showed that silver nanoparticles-loaded ETGFA had superior antimicrobial effects on *P. aeruginas, S. aureus* and *E. coli,* while *C. albicans* was less sensitive to it. It could probably be explained that *C. albicans* was a eukaryotic cell, whose electron transfer chain proteins are hidden in the intracellular mitochondrion, so it requires high concentration of silver nanoparticle to overcome the membrane block to come in contact with its mitochondria. Conversely, the other tested bacteria are prokaryotes, whose electron transfer chain proteins are on membrane surface and thus are easily inactivated by silver nanoparticles (Alt et al., 2004). Therefore, the silver nanoparticles exerted more effective inhibition to prokaryotic *P. aeruginas, S. aureus* and *E. coli* when compared to eukaryotic *C. albicans*.

### *4.6. In vivo* irritation test

Potential irritation would be caused by drug accumulation at the contacting sites during application of vaginal formulation. The blank ETGFA showed no significant toxicity indicated by a comprehensive irritation index of 0 based on the control group of saline and the silver nanoparticle-loaded ETGFA showed much slighter irritation with comprehensive irritation index at 2.66 than that of silver nanoparticle solution with comprehensive irritation index at 3.25. It can be concluded that the blank ETGFA caused no tissue irritation based on its comparison with saline and the blank ETGFA (*p* > .05, Table S8) and that the irritation of the silver nanoparticle-loaded ETGFA was mainly caused by the silver nanoparticles based on the comparison of the blank ETGFA and silver nanoparticle-loaded ETGFA (*p* < .01, Table S8). In addition, incorporation of silver nanoparticles in the ETGFA could reduce the irritation of silver nanoparticles as the irritation caused by the silver nanoparticle-loaded ETGFA was lower than that of silver nanoparticles with identical concentration.

The effective treatment and good biocompatibility was achieved by the appropriate drug concentration of ETGFA, which caused slight and tolerable stimulation to vaginal canal during administration. Additionally, foam softness reduced irritation associated with mechanic friction. The high loss on drying (>80 wt%) indicated reduced risk from drug carrier residence after effective treatment, avoiding the potential irritation to vaginal tissues.

## Conclusion

Vaginal drug delivery proves to be an optimal route for the local treatment of vaginal infection. Conventional vaginal drug delivery systems showed obvious limitations of short drug retention, indirect drug delivery and poor patient compliance, which hindered their application and development.

Herein, an expansible thermal gelling foam aerosol was designed to address these problems. For present formulation, there was a gelation process at physiological temperature after the administration. ETGFA could directly deliver silver nanoparticles to the deep vaginal rugae by foam expansion and its thermal sensitive gelation provided longer drug retention in vaginal canal owing to the adhesive strength and viscoelasticity. *In vitro* drug release and bacteria inhibitory tests indicated that the drug incorporation into ETGFA formulation achieved comparable drug release behavior to that of commercial gel Asimi^®^ with enhanced antimicrobial effects. The results indicated that ETGFA could be a candidate formulation for vaginal drug delivery.

## Supplementary Material

IDRD_Pan_et_al_Supplemental_Content.docx

## Appendix

Supplemental material includes Figure S1, Tables S1–S8 and detailed information about the preparation of stimulus vaginal fluid mentioned in the manuscript. Figure S1 shows adhesive strength of formulations with different adhesive agents. Tables S1 and S2 demonstrate the influence of the poloxamer concentration and SVF dilution on the gelation temperature. Tables S3 and S4 describe the MICs of both the silver nanoparticle-loaded ETGFA and silver nanoparticle solution on the four strains. Tables S5–S7 indicate the scoring details and the comprehensive results of irritation tests. Table S8 shows the detailed results of statistical analysis for the *in vivo* irritation test. The material is available free of charge via publisher.
