# Supplementary material for: Expansible thermal gelling foam aerosol for vaginal drug delivery
Source: Drug Deliv. 2017 Sep 18;24(1):1325–37. doi: 10.1080/10717544.2017.1375575 (PMC8241080; doi:10.1080/10717544.2017.1375575)
Supplement: IDRD_Pan_et_al_Supplemental_Content.docx [file IDRD_A_1375575_SM4429.docx]

**Expansible thermal gelling foam aerosol for vaginal drug delivery**

Liling Mei^1&^, Jintian Chen^1&^, Siqin Yu^1, 2&^, Ying Huang^1^, Yecheng Xie^1^, Hui Wang^1^, Xin Pan^1^*, Chuanbin, Wu^1^

^1^ School of Pharmaceutical Sciences, Sun Yat-sen University, Guangzhou 510006, China

^2^ Guangzhou Egg-Biotech Co.,Ltd., Guangzhou 510663, China

*Corresponding Author: Xin Pan, e-mail: pxin_1385@163.com

^&^ First Co-authors：These authors have contributed equally to this work.

**Supplemental materials**

# 1 Optimization of poloxamer concentrations to modulate the gelation temperature

Poloxamer 407 (P407) was utilized in the formulation as the basic thermal sensitive material while poloxamer 188 (P188) acted as a temperature modulator to obtain optimal gelation temperature. Various concentrations of P407 (18%, 20%, 22%, w/w) and P188 (0%, 3%, 5%, w/w) were applied to investigate their influence on gelation temperature. Gelation temperature was evaluated by a rotational rheometer with the same method as that of the drug-loaded ETGFA.

**Table S1. Effect of poloxamer concentrations on the gelation temperature (*n* = 3)**

| Formulation | P188  (wt%) | P407  (wt%) | Gelation temperature (°C) |
| --- | --- | --- | --- |
| 1 | 0 | 18 | 24.67 ± 0.58 |
| 2 | 3 | 18 | 31.00 ± 0.32 |
| 3 | 5 | 18 | 34.83 ± 0.41 |
| 4 | 0 | 20 | 22.17 ± 0.87 |
| 5 | 3 | 20 | 25.83 ± 0.28 |
| 6 | 5 | 20 | 29.16 ± 0.57 |
| 7 | 0 | 22 | 19.83 ± 0.29 |
| 8 | 3 | 22 | 22.16 ± 0.59 |
| 9 | 5 | 22 | 24.16 ± 0.20 |

Multiple linear regression analysis was conducted to statistically analyze the relationship of gelation temperature and poloxamer concentrations (Table S1) by Statistical Analysis System 9.4 software (SAS 9.4). And the best fitted equation (R^2^=0.9967, F=507.38, P<0.0001) was obtained as Formula S1:

*T*= 47.03404-1.24303*C*_P407_+7.32307*C*_P188_-0.2948*C*_P407_*C*_P188_ (Formula S1)

where *T* is the gelation temperature and *C* is the concentration of poloxamer.

The most appropriate gelation temperature of 34.83 ± 0.41°C could be achieved with 18 wt% P407 and 5 wt% P188 (Table S1). In order to obtain the abovementioned 18 wt% P407 and 5 wt% P188 after dilution, the concentrations of P407 and P188 in the pure ETGFA should be adjusted to 23.4% and 6.5%, respectively, with consideration of the dilution effect of vaginal fluid in a volume ratio of 10:3 (ETGFA: vaginal fluid). The gelation temperature of pure ETGFA with such poloxamer concentrations was 22.01 ± 0.62°C, lower than room temperature of about 25°C, which indicated that thermal gelation occurs at ambient temperature, causing obstacles for storage and administration of the formulation. In order to develop an optimal formulation which remains in the solution state at room temperature and thermally transforms into gel at physiological temperature, the concentration of P188 remained at 6.5% and the concentration of P407 was lowered to 20%, 21% and 22% to optimize the thermal response of the ETGFA. The results were shown in Table S2. The gelation temperature without SVF ought to be higher than the ambient storage temperature to remain in the solution state while the gelation temperature of the ETGFA diluted with SVF is supposed to be slightly lower than the body temperature, providing a swift phase transition from liquid foam to solid gel after administration.

**Table S2. The dilution effect of SVF on the gelation temperature (*n* = 3)**

| Formulation | P188 | P407  (wt%) | Gelation temperature (°C) | |
| --- | --- | --- | --- | --- |
|  | (wt%) |  | without SVF | with SVF |
| 1 | 6.5 | 20 | 32.8 ± 0.27 | 37.9 ± 0.35 |
| 2 | 6.5 | 21 | 28.8 ± 0.31 | 35.7 ± 0.26 |
| 3 | 6.5 | 22 | 26.9 ± 0.22 | 34.0 ± 0.41 |

For 20% P407 and 6.5% P188, the gelation temperature after dilution was difficult to achieve at physiological condition while for 22% P407 and 6.5% P188, the gelation temperature for storage was close to room temperature and may lead to undesired gelation before administration. Therefore, the poloxamer concentrations in the formulation of ETGFA was selected at 21% for P407 and 6.5% for P188 (Table S2) to obtain a favorable gelation temperature of pure formulation (28.8 ± 0.3°C) higher than room temperature and that of diluted formulation (35.7 ± 0.3°C) slightly lower than the physiological temperature.

# 2 Preparation of stimulus vaginal fluid (SVF)

SVF was prepared with ingredients as following: 3.51 g sodium chloride, 1.40 g potassium hydroxide, 0.22 g calcium hydroxide, 0.018 g bovine serum albumin, 2.00 g lactic acid, 1.00 g acetic acid, 0.16 g glycerol, 0.40 g urea and 5.00 g glucose per liter, with pH at 3.8~4.4.

# 3 Selection of adhesive agent

Optimal adhesive agent is supposed to be nontoxic and possess intensive adhesion to mucosa. In this study, 0.2 wt% adhesive agents including Arabic gum, sodium carboxymethyl cellulose (CMC-Na), sodium alginate, carbopol and xanthan gum, were screened. Adhesive strength including tensile stress and shear stress, were compared to select the optimal adhesive agent.


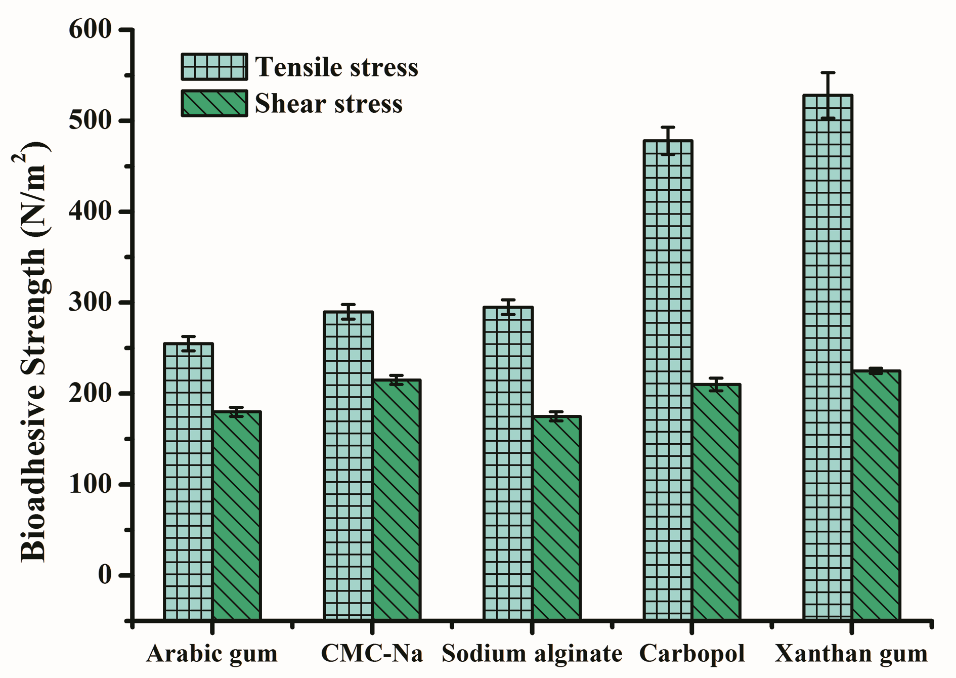


**Figure S1 Adhesive strength of formulations with different adhesive agents (*n* = 3)**

The results showed that there was no significant difference among shear stress while carbopol and xanthan gum had superior tensile stress than other adhesive agents. Xanthan gum had high tensile stress but it failed to achieve uniformly dispersion in base solution of the ETGFA. Carbopol was selected as the adhesive agent for its better dispersion.

# 4 MICs of the drug-loaded ETGFA and silver nanoparticles solution on *P. aeruginas, S. aureus, E. coli* and *C.albicans*.

The detail results of MICs (Table S3, S4) demonstrated that different floras of bacteria had varied sensitivities to silver nanoparticles. The growth of both *E. colis* and *S. aureus* was inhibited at 25.0 μg/mL. *P. aeruginasa* was more sensitive as its growth was inhibited at a lower concentration of 12.5 μg/mL. Contradictorily, *C.albicans* was more resistant to silver nanoparticles solution than the other bacteria with a higher MIC at 100.0 μg/mL. The MIC results for drug-loaded ETGFA were the same as that of the free silver nanoparticles solution, indicating that the incorporation of sliver nanoparticles into ETGFA did not attenuate the *in vitro* antimicrobial efficacy of silver nanoparticles.

**Table S3. MICs of silver nanoparticle-loaded ETGFA on *P. aeruginas*, *S. aureus*, *E. colis* and *C.albicans***

| Floras | Bacteria growth under different drug concentrations (μg/mL) | | | | | | | | |
| --- | --- | --- | --- | --- | --- | --- | --- | --- | --- |
|  | 200 | 100 | 50 | 25 | 12.5 | 6.25 | 3.125 | Positive control | Negative control |
| *E. colis* | - | - | - | - | + | + | + | + | - |
| *S. aureus* | - | - | - | - | + | + | + | + | - |
| *p .aeruginasa* | - | - | - | - | - | + | + | + | - |
| *C.albicans* | - | - | + | + | + | + | + | + | - |

“-”indicates no colony observed；“+” indicates colonies observed.

**Table S4. MICs of silver nanoparticles solution on *P. aeruginas*, *S. aureus*, *E. colis* and *C.albicans***

| Floras | Bacteria growth under different drug concentrations (μg/mL) | | | | | | | | |
| --- | --- | --- | --- | --- | --- | --- | --- | --- | --- |
|  | 200 | 100 | 50 | 25 | 12.5 | 6.25 | 3.125 | Positive control | Negative control |
| *E. colis* | - | - | - | - | + | + | + | + | - |
| *S. aureus* | - | - | - | - | + | + | + | + | - |
| *p .aeruginasa* | - | - | - | - | - | + | + | + | - |
| *C.albicans* | - | - | + | + | + | + | + | + | - |

“-”indicates no colony observed；“+” indicates colonies observed.

# 5 Irritation scores of vagina caused by ETGFA

Specimens from vagina, ovary and uterus were rated and scored for the irritation evaluation. Score *S* of tissue irritation in each rat was calculated according to Formula S2:

$S=\frac{\sum_{A}^{D} vagina+\sum_{A}^{D} ovary+\sum_{A}^{D} uterus}{3}$ (Formula S2)

where *S* is the comprehensive irritation index and A~D is the score of each tissue shown in Table S5.

The scoring standard referred to Table S5.

**Table S5. Evaluation standard of irritation score in histopathological specimens**

| Tissues | Rated items and extent | Details | Score |
| --- | --- | --- | --- |
| A Endothelial tissue | Normal | Intact | 0 |
|  | Extremely light | Cell degeneration or flat | 1 |
|  | Slight | Organization deformation | 2 |
|  | Moderate | Local erosion | 3 |
|  | Severe | Spread erosion | 4 |
| B Leucocyte infiltration | Normal | 0 | 0 |
|  | Extremely light | 1~25 | 1 |
|  | Slight | 26~50 | 2 |
|  | Moderate | 51~100 | 3 |
|  | Severe | > 100 | 4 |
| C Vascular congestion | Normal | 0% | 0 |
|  | Extremely light | 1%~20% | 1 |
|  | Slight | 21%~40% | 2 |
|  | Moderate | 41%~60% | 3 |
|  | Severe | 61%~100% | 4 |
| D Edema | Normal | 0% | 0 |
|  | Extremely light | 1%~20% | 1 |
|  | Slight | 21%~40% | 2 |
|  | Moderate | 41%~60% | 3 |
|  | Severe | 61%~100% | 4 |

*For leucocyte infiltration, the number indicates the number of leucocytes under the microscopy of 400 magnification times;

The irritation scores of four rats in each test group treated with saline solution, blank ETGFA, drug-loaded ETGFA and silver nanoparticles solution, respectively, were shown in Table S6. Comprehensive irritation index (CII) was calculated by the mean scores minus that of control group (saline solution). It was indicated by the irritation index that blank ETGFA caused no irritation to the vagina. The irritation induced by drug-loaded ETGFA was extremely slight (CII = 2.66) while that of silver nanoparticles solution was a little severe (CII = 3.25) compared to saline control group. The aforementioned results demonstrated that the irritation caused to the disease site was tolerable and the incorporation of silver nanoparticles into ETGFA did not cause additional irritation.

**Table S6. Specific scores of each rat for irritation test**

| Groups | Number of rats | irritation scores |
| --- | --- | --- |
| Saline solution | 1 | 0.33 ± 0.44 |
|  | 2 | 1.00 ± 0.00 |
|  | 3 | 1.00 ± 0.00 |
|  | 4 | 0.33 ± 0.44 |
| Blank ETGFA | 1 | 0.33 ± 0.44 |
|  | 2 | 1.00 ± 0.00 |
|  | 3 | 0.00 ± 0.00 |
|  | 4 | 1.33 ± 0.44 |
| Drug-loaded ETGFA | 1 | 2.33 ± 0.44 |
|  | 2 | 3.33 ± 0.89 |
|  | 3 | 4.67 ± 0.89 |
|  | 4 | 3.00 ± 0.00 |
| Silver nanoparticles solution | 1 | 3.33 ± 0.89 |
|  | 2 | 4.33 ± 0.44 |
|  | 3 | 4.67 ± 0.44 |
|  | 4 | 3.33 ± 0.44 |

**Table S7. Comprehensive irritation scores for the treated groups (*n* = 4)**

| Group | Group score | Irritation index* |
| --- | --- | --- |
| Saline solution | 0.67 ± 0.38 | / |
| Blank ETGFA | 0.67 ± 0.61 | 0.00 |
| Drug-loaded ETGFA | 3.33 ± 0.98 | 2.66 |
| Silver nanoparticles solution | 3.92 ± 0.69 | 3.25 |

*Vaginal irritation is indicated by comprehensive irritation index (CII) as the following standard: normal (CII < 1), extremely light (1 < CII≤ 5), slight (5 < CII ≤ 9), moderate (9 < CII≤12) and severe (12<CII).

**Table S8. Statistical Analysis of postoperative analgesic effect (*n* = 4).**

| Time  (h) | Saline  solution | Blank ETGFA | Ag-NP loaded ETGFA | Ag-NP solution |
| --- | --- | --- | --- | --- |
| Blank ETGFA | ns |  |  |  |
| Ag-NP loaded ETGFA | ****** | ****** |  |  |
| Ag-NP solution | ******* | ******* | ns |  |

^ns^ *P* > 0.05, **^*^** *P* < 0.05, **^**^** *P* < 0.01, **^***^** *P* < 0.001. Ag-NP indicates Silver nanoparticle.
